# Is the Injectable Contraceptive Depo-Medroxyprogesterone Acetate (DMPA-IM) Associated with an Increased Risk for HIV Acquisition? The Jury Is Still Out

**DOI:** 10.1089/aid.2019.0228

**Published:** 2020-04-30

**Authors:** Janet P. Hapgood

**Affiliations:** ^1^ Department of Molecular and Cell Biology, University of Cape Town, Cape Town, South Africa.; ^2^ Institute of Infectious Disease and Molecular Medicine, University of Cape Town, Cape Town, South Africa.

**Keywords:** Depo-Provera, DMPA, contraception, HIV, ECHO trial

## Abstract

Intramuscular depo-medroxyprogesterone acetate (DMPA-IM) is the most widely used hormonal contraceptive in sub-Saharan Africa. Previous meta-analyses of observational studies found a significant 40%–50% increased risk associated with DMPA-IM use, relative to no contraception or infrequent condom use. This raised substantial concerns, although these studies had important limitations. Consequently, the open-label randomized Evidence for Contraceptive Options and HIV Outcomes trial was conducted, designed primarily to detect a 50% or greater difference in HIV risk between DMPA-IM, the levonorgestrel (LNG) implant, and the copper-intrauterine device. The ECHO study, published in July 2019, concluded that there is no substantial difference in HIV risk among the methods evaluated, and that all three methods are safe and highly effective. In response, the WHO relaxed the Medical Eligibility Criteria for DMPA-IM use among women at high HIV risk in August 2019. However, two of the three comparisons in the ECHO trial could rule out neither a 50% increase nor no change in HIV risk for one contraceptive compared with another. The study had limitations and the results contained considerable uncertainty. They also did not inform on associated HIV risk for any one of the individual methods due to the absence of a control group such as no contraception or only infrequent condom use. The HIV risks associated with LNG implant and copper-IUD relative to no contraception or infrequent condom use are unknown and these cannot be seen as controls, nor did the authors claim them to be. The results will be discussed in the context of their limitations, what they add to the body of work to date on contraception and HIV acquisition, and the implications of the findings and reports thereof for future research and contraceptive choice.

## Introduction

About 38% (16.5 million) of all contraceptive users in sub-Saharan Africa use progestin-only injectable hormonal contraceptives (HC).^1^ Depo-medroxyprogesterone acetate (DMPA), a three-monthly, intramuscular (IM) injectable HC,^2^ is the most commonly used HC in sub-Saharan Africa and South Africa.^3^ Two meta-analyses of high-quality observational studies found a significant 40%–50% increased risk associated with DMPA-IM use compared with women not using hormonal contraception^4,5^ [adjusted hazard ratio, aHR 1.50 (95% confidence interval, CI 1.24–1.83),^5^ aHR 1.40 (95% CI: 1.23–1.59)^4^]. However there remains uncertainty about whether the increased risk of HIV acquisition was a “real effect of the contraceptive method used or whether it was a statistical artefact resulting from key limitations of observational studies (residual confounding in particular),” as quoted from a 2019 WHO report.^6^ The ECHO trial was conducted primarily in response to these substantial concerns regarding DMPA-IM and HIV risk, as indicated by their “background” statement.^7^ The ECHO trial was an open-label randomized trial designed to detect whether there is a difference in HIV risk between three contraceptive methods, that is, DMPA-IM, levonorgestrel (LNG) implant, a progestin-only implant containing LNG, and a copper-IUD, a copper-containing non-hormonal intrauterine device.^7^

The planning of the ECHO trial received wide publicity and raised expectations that it would provide clear answers on whether DMPA-IM is associated with increased risk of HIV acquisition. The planned study was controversial regarding the ethics of the trial and whether the results would likely be conclusive.^8–10^ The results were published online on June 13th, 2019 in *The Lancet.*^7^ The trial was well conducted and achieved a remarkable level of adherence and participation of 7,928 girls and women and involved multiple sites and stakeholders. A major benefit of the trial was strengthening of networks and capacity building across disciplines and highlighting and raising awareness of the high HIV incidence in the study areas, although the latter was already well publicized before the trial. The authors summarized their findings as follows: “We did not find a substantial difference in HIV risk among the methods evaluated, and all methods were safe and highly effective. HIV incidence was high in this population of women seeking pregnancy prevention, emphasising the need for integration of HIV prevention within contraceptive services for African women. These results support continued and increased access to these three contraceptive methods.”^7^

The ECHO results are likely to have an enormous impact on regional and international public health and clinical practices and, thus, warrant detailed discussion and close scrutiny. Primarily in response to the ECHO results, updated WHO guidelines were published on August 29th, 2019, changing the Medical Eligibility Criteria (MEC) for DMPA-IM use among women at high risk of HIV infection from category 2 to category 1 (advising use of the method in any circumstances).^6^ An article that argues that the trial was unethical was also published.^11^ Initial press releases and media reports focused on DMPA-IM and, in my view, largely misreported or misinterpreted the ECHO results.^12–19^ This commentary will focus on the ECHO trial results regarding HIV risk and DMPA-IM and the interpretation, reporting, and broader implications thereof.

## What Do the ECHO Results Tell Us About Relative HIV Risk for the Three Methods?

Results presented in the ECHO paper^7^ for two of the three comparisons for relative HIV risk (Table 1) fell into a “grey zone,” that is, they were inconclusive since the CIs for these comparisons included values of both 1.0 and 1.5 and, thus, could rule out neither a difference in risk nor a 50% increase in risk for one contraceptive compared with another. Understanding whether any contraceptive method was associated with a 50% increase in HIV acquisition relative to any of the other methods was one of the major goals of the study. The ECHO study was designed to detect a difference between HIV risk of 50% or more between any two of the three methods of contraception. It would seem reasonable to say that the difference in HIV risk is not 50% or more between DMPA-IM and copper-IUD, because the CI excludes the values 0.5 and 1.5. However, these results are also compatible at the 96% confidence level with an 18% decreased risk and a 44% increased risk. There is less certainty regarding whether a difference was or was not apparent between DMPA-IM and LNG implant, or copper-IUD and LNG implant. For copper-IUD versus LNG implant, one could conclude that differences from a 10% decreased HIV risk to a 55% increased risk are compatible with the data at the 96% confidence level, with the point estimate of an 18% increased risk associated with copper-IUD compared with the LNG implant. The results for DMPA-IM versus LNG implant are compatible with a minimal 2%–5% decrease to a 59%–71% increased risk at the 96% confidence level with the point estimate of increased risk associated with DMPA-IM of 23%–29% compared with the LNG implant.

**Table 1.** ECHO Trial Results: Hazard Ratios for HIV Acquisition

Analysis	DMPA-IM vs. copper-IUD		DMPA-IM vs. LNG implant		Copper-IUD vs. LNG implant	
	HR (96% CI)	p	HR (96% CI)	p	HR (96% CI)	p
Intention-to-treat	1.04 (0·82–1·33)	.72	1.23 (0.95–1.59)	.097	1·18 (0·91–1·53)	.19
Causal/continuous	1.10 (0·84–1·44)	.49	1.29 (0·98–1·71)	.060	1·18 (0·90–1·55)	.22

CI, confidence interval; DMPA-IM, intramuscular depo-medroxyprogesterone acetate; HR, hazard ratio; LNG, levonorgestrel.

The study could thus neither prove nor rule out a finding of a 50% increase in HIV acquisition for DMPA-IM versus the LNG implant or for the copper-IUD versus LNG implant. It is correct to say that a point estimate of at least a 50% increased risk was not detected for two of the three comparisons, but it is not correct to conclude that at least a 50% increased risk does not occur for two of the three comparisons, or that there is no difference in risk for any of the three comparisons. In this case, absence of definitive evidence of a 50% increased risk between groups is not evidence of absence of an effect.^20^ The authors do not directly make such incorrect statements, although some of their general conclusions imply this and unfortunately this is how the data have been misinterpreted by many, as discussed in the Messaging: Challenges, Misreporting and Consequences section.

Interpretation of the ECHO results raises important questions about statistical analysis of data and interpretation of *p*-values, a topic that has been recently highlighted in the literature.^21–26^ Whether or not an effect is “detected” or “shown” is usually based on whether a *p*-value is less than a cut-off representing the maximum acceptable false-positive (“Type-I”) error rate, usually defaulted to .05 (or .04 for the case of ECHO). The choice of a 96% and not a 95% confidence level for the primary comparisons is explained by the authors as follows: “The type I error was chosen to control the family-wise error rate for the three HIV endpoint comparisons at 0 · 10; thus, each of the three comparisons was planned to be assessed with a two-sided type I error rate of 0 · 04 (and corresponding 96% CIs).”^7^ Equivalently, the same conclusion is based on whether the corresponding CI excludes a value of 1, in the case of the primary analysis in the ECHO trial. Depending on how this is interpreted, there is great potential for misleading conclusions. Amrhein *et al.* explain the danger of false conclusions by giving examples of data very similar to some data from the ECHO trial.^22^ Further, the ECHO trial was designed as a superiority study that can detect differences between methods but cannot prove equivalence, or no difference between methods. Statisticians have warned against making “yes/no” decisions based on *p*-values or CIs.^22^ For example, although the causal-continuous analysis of the DMPA-IM versus LNG implant showed a best estimate of a 29% increased risk with a *p*-value of .06 (significance level 0.04) and the lower limit of the CI of 0.98 [1.29 (0.98–1.71)], this appears to have been interpreted as lack of evidence of a likely association. However, another comparison [1.14 (1.00–1.30), *p* = .057; significance level 0.05] is reported in the appendix of *The Lancet* article^7^ as evidence that “HSV-2-positive status was associated with higher HIV incidence than HSV-2-negative status,”^7^ showing how subjective such decisions can be.

Interpretation of 50% versus 30% values in the ECHO trial as limits of detectable difference is also confusing and possibly misleading. Based on the ECHO pre-trial power analysis,^7^ it was theoretically possible to conclude that a 50% increased risk was or was not detected. However, it cannot be presumed that if such a difference was not detected as judged by a *p*-value, that a difference of 50% or more does not occur.^27,28^ The ECHO authors further stated that “under the design of this study an observed approximately 30% increase in HIV incidence would have been found to be statistically significant and HRs less than approximately 1.17 would have upper limits of the 96% CIs that would have ruled out a 50% increase.”^7^ This statement appears to be based on a *post hoc* power analysis. *Post hoc* power analyses are contentious and have been argued to have little value.^28^ This should not be taken as proof that the data show that an effect >30% does not occur.

Importantly, the ECHO results raise concerns about the relative safety regarding HIV risk for DMPA-IM compared with the LNG implant and the data contain considerable uncertainty. They also raise the possibility based on two of the three comparisons that the LNG implant is a safer alternative than the other two methods in terms of HIV susceptibility.

## What is a “Substantial” or “Meaningful” Difference in HIV Risk?

The ECHO results raise important issues regarding how and by whom an acceptable level of risk for a contraceptive is assessed, communicated, and used to inform policy decisions. The use of words such as “substantial” or “meaningful” in the ECHO article are highly subjective and can be misleading. As mentioned in the Introduction section, the authors concluded that: “We did not find a substantial difference in HIV risk among the methods evaluated, and all methods were safe and highly effective.”^7^ “Substantial” in this context could be interpreted in many ways, including to mean “50% or more,” “important,” “real,” or “significant.” Elsewhere the authors state that: “We chose a 50% increase in HIV risk on the basis of formative work with stakeholders to determine a meaningful difference that would inform policy change.”^7^ Thus, even if by “substantial” it is meant “50% or more,” the basis for a 50% threshold, a very important decision with enormous public health implications, is not explained. The article referenced for this decision^29^ does not discuss how a value of 50% was calculated or estimated. If this was based on wanting to test whether observational data showing a best estimate of a 50% increased risk associated with DMPA-IM versus no contraception or mainly condom usage^5^ was true—the most rigorous and concerning clinical data published at the time that largely justified the ECHO study—then the decision is problematic. This is because the ECHO trial could be predicted to likely not detect at least a 50% effect for DMPA-IM relative to the other two methods, unless both of them had zero associated HIV risk relative to no contraception. The latter is highly unlikely. For example, if both the LNG implant and the copper-IUD are associated with only a 10% increased HIV risk relative to no contraception, and DMPA-IM is associated with a 50% increased HIV risk relative to no contraception, the risk for DMPA-IM relative to both methods would be 36%, that is, well below the 50% threshold.

The authors also make the statement on page 8 that “Although this trial had low statistical power to detect an increase in HIV incidence of less than 30%, for individual women at very high HIV risk, we acknowledge that even a relatively small effect might be important in contraceptive and HIV prevention decision making.”^7^ This statement seems at odds with the reasons given for the choice of 50% and seems to be largely ignored in the rest of the article and particularly in the communications of the study results. If the choice of the 50% threshold was based on financial considerations to do with affordability of the trial (higher power to detect smaller differences would have required more participants and been more expensive), this should be acknowledged rather than suggesting that an effect of less than 50% would not be “substantial” or “meaningful” or sending mixed messages.

What we do know is that there were about 980,000 new HIV infections in sub-Saharan Africa in 2017 alone, with 59% of these occurring in women.^30^ We also know that about 33% of all new infections in eastern and southern Africa (800,000 new infections in 2017) occurred in South Africa, which has about 7.7 million people living with HIV, with HIV incidence in some areas being extremely high (4.7–15.2 per 100 woman-years).^5,30^ “HIV hotspots” occur in South Africa where 40.8% (39.5%–42.1%) of adults (aged 15 years and older) are living with HIV.^30^ Worldwide, high HIV prevalence correlates with high usage of injectable HC.^31^ DMPA-IM is the most commonly used HC in sub-Saharan Africa and South Africa.^3^ In 2016, about 42% of sexually active women aged 15–49 that used any modern method of contraception in South Africa used injectable contraceptives, whereas only about 2% of these used some form of IUD (hormonal or non-hormonal) and 7% used an implant.^32^ Of all sexually active women, 25% used injectables in 2016 in South Africa.^32^ Based on available data,^30,32^ one can estimate that of the ∼155,760 new infections in sexually active women in South Africa in 2017, at least 27,258 were likely on DMPA-IM. If DMPA-IM increases HIV risk relative to no contraception use by 25% or 50%, this could account for 5,387 or 10,340, respectively, of new HIV infections in women and girls due to DMPA-IM usage per annum, just in South Africa.

It is doubtful that individual women and girls and health care providers would consider these potential increased risks as not being “substantial” or “meaningful.” The use of such words needs to be explained and justified in the context of prevalence and incidence of an effect. In support of this argument, it is interesting to note that outcomes from a Guideline Development Group (GDG) in the 2019 WHO MEC document^6^ include the statements: “The community stakeholder presentation emphasized that, for some women, any level of increased HIV risk would be too high. It also highlighted that the ECHO trial was not set up to assess the difference in the risk of HIV acquisition between contraceptive users and non-users.” A recent modeling paper concluded that if there is a 20% increased risk of HIV-1 acquisition associated with DMPA-IM use in girls and women, compared with no associated increased risk for DMPA-IM, this could have substantially increased the scale of the HIV epidemic in South Africa, affecting not only the users of DMPA-IM but also their male partners and the wider population.^33^ This article highlights the importance of carefully considering the future consequences of contraceptive policy choices, even for increased HIV risks of 20%.

## What Were the Limitations of the ECHO Trial?

As discussed in detail earlier, the study had low power to detect differences below 50%, was not able to conclude that no differences occurred between methods and there was considerable uncertainty in the data as indicated by the CIs including both the null hypothesis (value of 1) and a 50% difference for two of the primary comparisons. The ECHO study was a randomized clinical trial, not a randomized controlled trial, that is, there was no suitable control group (placebo or no contraception or infrequent condom use only). Besides these limitations, there were several other limitations and sources of uncertainty.

In general, open-label randomized trials (randomized before initiation) have limitations due to potential confounding factors, since randomization only applies before initiation of treatment and bias can occur post-initiation, once participants, clinicians, counseling staff, and researchers know to which arm of the study participants are assigned.^34^ Possible confounding factors include incorrect self-reporting of condom usage and sexual behavior (frequency of intercourse etc.).^5^ Women participating in HIV acquisition and contraception trials are counseled to use condoms, but if they all do so during intercourse, then there would be almost no new HIV infections and no conclusions would be possible regarding HIV risk. Women are asked to self-report their condom usage and sexual behavior, which is corrected for in observational studies and in open-labeled studies after initiation if using causal analysis. Thus non-random inaccuracies in self-reporting of condom usage had the potential to confound^35^ the ECHO data post-initiation. In a trial that is not randomized before initiation, it is likely that such confounding factors would be greater during the trial than in a trial that is randomized before initiation, as the groups in each study arm could have different characteristics related to the chosen method of contraception. However, it cannot be claimed that potential confounding factors disappeared in the ECHO trial after initiation. Women could have exhibited non-random degrees of correct reporting of condom usage, sexual behavior, and/or additional non-study contraception use, resulting in bias unrelated to biological or inherent effects of the assigned method, but rather related to being on the trial.

Some bias may have been due to unequal counseling or perceptions of risk. This seems likely given that concerns regarding DMPA-IM and HIV risk were widely publicized before and during the trial, unlike for the other two arms, and the trial involved an exceptionally high degree of counseling.^6^ This could have been even greater than during previous observational trials, especially since the MEC for DMPA-IM was changed from 1 to 2 in 2016 during the ECHO trial, suggesting increased counseling for DMPA-IM users, due to increased concerns regarding HIV risk after publication of the meta-analysis data.^5^ The authors argue that bias related to behavioral changes due to method-related counseling or differential reporting was unlikely to have affected their results. However, this is impossible to monitor accurately, cannot be discounted as a possibility, and is an important limitation of the ECHO open-label trial.

As is the case for most clinical trials, the different methods of data analysis used by the authors in the ECHO study have inherent limitations and uncertainties.^34^ A limitation of the intention-to-treat (ITT) analysis is that it does not correct for any known (such as differences in sexual behavior and discontinuation of a method in the case of the ECHO trial) or unknown non-random pre- or post-initiation confounding effects. A limitation of the causal-continuous method of analysis is that it performs weighting and adjustments based on only measured observational factors, some of which are self-reported and subject to error. More uncertainty can sometimes also be introduced due to lowered power (fewer women as some are excluded). In general, ITT analysis can underestimate the real effect of a treatment if there are confounding factors, whereas the causal-continuous method of analysis can give a truer reflection of the biological effect. Which method of analysis gives the truest reflection of an effect appears to be controversial and to depend on the particular trial.^34^ In a blinded randomized trial, ITT analysis is generally good at overcoming residual confounding bias, but in an open-label unblinded trial such as the ECHO trial, the advantage is less clear.

Results for the causal-continuous analysis reported in the appendix of *The Lancet* article^7^ show that after weighting and adjusting for baseline and time-varying covariates, the point estimate moved from 23% (ITT) to 29% (causal-continuous) and the *p*-value from .09 (ITT) to .06 (causal-continuous) for the DMPA-IM versus LNG implant.^7^ Interestingly, after adjustment for baseline covariates, but without weighting, the point estimate and *p*-value moved in the same direction (34% and .029, respectively) and the CI excluded 1.00, a result that the authors state is “statistically significant.”^7^ The relevance of the causal-continuous analysis results for DMPA-IM is glossed over in the manuscript and is not reported in the abstract. The causal-continuous analysis results strongly suggest that DMPA-IM increases HIV risk compared with the LNG implant by about 29%–34%, and cannot exclude an effect as large as 77%, at the 96% confidence level. If DMPA-IM is associated with a 40% increased HIV risk and the LNG implant with a 10% increased risk, then the increased risk for DMPA-IM relative to LNG implant would be 27%, within the 23%–29% range of the point estimates reported by the ECHO authors. Although the ECHO results obviously do not prove that DMPA-IM is associated with a 40%–50% increased HIV risk, they also do not disprove this nor do they provide the basis for a strong argument that the prior observational analyses comparing DMPA-IM with no hormonal use were confounded and inaccurate.

Another limitation of the ECHO data was the potential for non-random baseline and post-initiation non-study progestin exposure between arms. Although the authors detected similar levels of unreported DMPA-IM usage pre-initiation in about 11%–14% of participants for each study arm, this was assumed after extrapolating from serum measurements on a subgroup of about 8% of women in each study arm. These women were also not excluded or corrected for in either method of data analysis. It is also unknown which other unreported progestins the participants were exposed to pre-initiation. Results showing misreporting of contraceptive hormone use in clinical research participants were recently reported by Achilles *et al.*^36^ They found that 27% of women using the same three contraceptive methods as in the ECHO trial and who reported no contraceptive use at baseline had objective evidence of hormonal contraception use at enrolment. In addition, 36% had objective evidence of non-study hormonal use at any time post-initiation, with the effects being unequal between study arms.^36^ Many of the latter were on LNG-containing contraceptives, most likely including some on combined oral contraceptives containing LNG and estrogens. Some of the effects just described could be inherent to the contraceptive method, but in the ECHO trial some could be non-random and related to the trial, where women were randomized to a method they would not necessarily have chosen outside the trial and most would not have been taking before the trial. If this occurred in the ECHO trial, it could have introduced bias.

It should further be noted that the relative risks reported in the ECHO trial only apply to a period of 18 months and the relative risks for longer duration of use are unknown. It is clear from the published data in *The Lancet* article^7^ that cumulative probability of HIV acquisition increases over time for all methods. However, the data suggest that differences between methods become more apparent at 18 months compared with 12 and 15 months,^7^ suggesting that they may have been even greater at 24 months.

It is self-evident that no clinical trial, including the ECHO trial, is perfect. There were several limitations to the ECHO trial, including known and unknown confounding factors. The concern is that several of the limitations just described were not clearly acknowledged in *The Lancet* paper,^7^ and none is mentioned in the communication of the results (see Messaging: Challenges, Misreporting and Consequences section). The ECHO trial results need to be interpreted with more caution, acknowledging alternative interpretations and in the context of their uncertainties and limitations.

## What Do the ECHO Results Tell Us About the Risks of HIV Acquisition Associated with DMPA-IM?

The ECHO results do not tell us anything about the absolute risk of HIV infection (relative to no contraception or mainly condoms), associated with any of the individual methods. The trial was not designed to address this question. The results do not resolve the controversy or provide more robust data or greater scientific certainty on this question, which has caused concern for decades. Changing the MEC for DMPA-IM from category 2 to 1 for women at high risk of HIV appears to be based primarily on one study, that is, the ECHO trial, which does not address the question of absolute HIV risk. No information can be inferred from the ECHO data on this question, because we do not know what the risk is for any of the three methods relative to no contraception or only condom use. In theory, each particular method could increase, have no effect, or decrease risk. The trial was not a controlled trial, that is, there was no placebo or control for no contraception or only condom use. The authors do not claim that their data inform on the risk of HIV associated with a particular method relative to no contraception. Further, although the WHO claims to have reviewed new biological data and found it to be “sparse,” “contradictory,” and of unknown clinical relevance, these conclusions are unsubstantiated and are not supported by any references to accumulated new data since 2016.^6^ Since 2016, there is substantial new clinical and *in vitro* biological and animal data that are largely consistent with increased HIV acquisition for DMPA-IM users via several mechanisms.^37–52^

An interesting argument was made in the WHO 2019 guidelines^6^ on this issue: “The GDG recognized that the ECHO trial did not address the etiological or causal question of whether DMPA increases the risk of HIV acquisition when compared with not using any contraception” followed shortly thereafter by: “Furthermore, the GDG noted that the high incidence of HIV infection experienced by each contraceptive group during the ECHO trial was similar to the background incidence assumed when designing the trial. This was deemed to be indirect evidence addressing the question, suggesting no increased risk of HIV acquisition among users of these contraceptives compared with women not using any contraception.” This argument is flawed. The “background incidence” assumed when designing the trial could not possibly have been accurate at the level required to make this conclusion. It was apparently based on multiple sources of data, including HIV incidence in these areas in women participating in HIV prevention trials,^29^ which would have included women on DMPA-IM. Another unknown variable, impossible to predict, is to what extent the high level and frequency of counseling, known to decrease HIV incidence, during the ECHO trial would have affected the “background incidence.”^53^ This point is also reinforced by the statement in the 2019 WHO guidelines that: “The high incidence of HIV was particularly striking given the extensive efforts made during the ECHO trial to provide HIV prevention counselling and interventions.”^6^ The WHO argument is ironic given that any such estimated value for “background incidence” would most likely involve a much higher degree of uncertainty than meta-analyses results based on decades of observational studies on DMPA-IM and HIV risk.

Whether LNG implant or copper-IUD increases HIV risk relative to no contraception or condoms only is unknown, due to very limited and uncertain data.^4,6,54^ There are published clinical and animal data that suggest plausible biological mechanisms whereby LNG, as well as MPA, may increase HIV acquisition by decreasing the integrity of the female genital tract barrier.^40–42,55^ The LNG implant was chosen over the etonogestrel implant for the ECHO trial due to its wider usage in Africa, although it is not reportedly less hypo-estrogenic than etonogestrel,^56^ as the authors claim in the Introduction section.^7^ The mechanism whereby the copper-IUD acts as a contraceptive is unclear but appears to be predominantly via inactivation of sperm and inflammation, due to release of copper ions.^57^ However, it is also theoretically possible that copper ions could inhibit HIV activity.^58^ Increased inflammation and sexually transmitted infections (STIs) are associated with increased HIV risk.^41^ Clinical data show that the copper-IUD has proinflammatory effects in the female genital tract, suggesting a plausible biological mechanism whereby the copper-IUD could also increase HIV acquisition via increased recruitment and activation of HIV target cells and/or disruption of epithelial barrier integrity.^41,59–62^ As for the LNG implant, it would be incorrect to assume or infer that the copper-IUD arm can be considered as a control.

Unsuitability of copper-IUD as a control with no effects on HIV risk is also reinforced by concerns regarding its inflammatory effects indicated by its current classification in the WHO MEC risk 2/3/4 categories for initiation for women at increased risk for STIs. MEC risk categories 1–4 refer to the following, respectively: (1) “Use the method in any circumstances,” (2) “Generally use the method,” (3) “Use of the method not usually recommended unless more appropriate methods are not available or not acceptable,” and (4) “Method not to be used.”^54^ Interestingly, the WHO changed the MEC for the copper-IUD from 2 to 1 for women at high risk of HIV infection, but not for high risk of STIs, in August 2019.^6^ This changed decision appears to be based on the absence of evidence for an association with HIV risk from one new low-quality, underpowered, observational study and from the ECHO results, which do not address the question.^54^

It would be incorrect to assume that women using copper-IUDs, or any other non-hormonal IUDs, make up a significant proportion of the control arms in the observational studies used in the meta-analyses.^4,5^ The control groups for these observational studies either excluded women using any form of contraception, including non-hormonal intrauterine devices,^63,64^ or included some women using non-hormonal contraception. The latter include different forms of non-hormonal intrauterine devices that typically, however, together made up only about 1% of the control group in some studies.^65^ This is consistent with the very low usage of non-hormonal intrauterine devices in the study areas.^32,65^ For example, at most 1% of all women in 2016 in South Africa used any form of IUD (hormonal or non-hormonal).^32^ The control group in observational studies using non-hormonal contraception typically consists of about 44% women on no form of contraception and about 50% women using condoms only, most of whom are using condoms inconsistently.^65^ In the absence of clarity on this point, some may mistakenly infer that the ECHO result for DMPA-IM versus copper-IUD^7^ can be compared with the observational meta-analysis data for DMPA-IM versus control,^4,5^ proving the latter to not be causal and to be confounded, as “proven” by higher quality data from the ECHO trial.

It would also be unscientific to infer that if a difference of at least 50% in HIV risk was not detected for DMPA-IM versus copper-IUD, the progestin MPA cannot be increasing risk of HIV acquisition. There is a large body of evidence showing that multiple mechanisms are likely to have an effect on HIV acquisition, including effects on immune function, inflammation, barrier integrity, and the microbiome.^41^ Each of the three methods could theoretically contribute more or less to any one or more of multiple mechanisms and result in any number of end-point differences in HIV risk due to a causal relationship.

The choice of LNG implant and copper-IUD as two of the three arms in the ECHO trial is perplexing, given the site locations and focus on South Africa (9/12 sites located in South Africa). Neither the copper-IUD nor the LNG implant has ever been widely used in South Africa, where there is limited use of an etonogestrel-containing implant. ECHO results for the LNG implant cannot be extrapolated to other methods, including use of LNG in oral contraceptive pills containing estrogen or LNG delivered intravaginally, or etonogestrel implants, due to different pharmacokinetics, doses, and/or progestins. Limited observational clinical data on HIV risk and laboratory data for the two-monthly injectable contraceptive norethisterone enanthate (NET-EN) do not raise concerns about an increased risk for HIV acquisition,^4,5,38,39,66–68^ unlike results for MPA.^38,39,41,52,67^ In observational studies, DMPA-IM use was significantly associated with increased HIV acquisition compared with NET-EN use [aHR 1.32, (95% CI 1.08–1.61)],^5^ a result very similar to the DMPA-IM versus LNG implant comparison in the ECHO trial. Why NET-EN was not chosen as one study arm in the ECHO trial is hard to understand, given that it is widely used in South Africa.^32^

## Messaging: Challenges, Misreporting, and Consequences

It is difficult to translate scientific results into clear but scientifically correct messages for a non-scientific audience. This is especially the case for the complex and widely anticipated ECHO trial results that had raised expectations and have impacted international public health policy, which will impact clinical practice and the health of millions of girls and women at high risk of HIV. It is, thus, particularly important to avoid reporting or implying false conclusions. Unfortunately, this has been the case for the ECHO trial. Most press releases and media articles have misreported or misinterpreted the results of the ECHO trial. The most misleading statements directly imply that the ECHO trial has shown that DMPA-IM is not associated with increased HIV risk relative to no contraception.^12–16^ Times Live reported that: “The most popular contraceptive injection in SA does not increase the risk of HIV, answering a question that has bothered scientists for decades”.^12^ *The Daily Maverick*, an influential online publication in South Africa, stated the following: “—more than 30 years after these issues were first raised—initial results of the Evidence for Contraceptive Options & HIV Outcomes study, known as the ECHO study, found no link between the risk of contracting HIV and the use of injectable contraception.”^16^ A WHO online publication “WHO Sexual and Reproductive Health” stated on June 13th, 2019 that “New study finds no link between HIV infection and contraceptive methods.”^17^ Many of the reports misinform by stating or implying that the ECHO trial has proved that DMPA-IM does not increase HIV risk relative to other methods, or has shown that there is no difference in risk between methods, or words to that effect.^17–19^ Almost all reports do not mention the uncertainty in the data or limitations of the ECHO trial, and most imply that DMPA-IM has been proven to be safe regarding HIV risk. Only one report mentions that the data suggest that the LNG implant is associated with lower HIV risk than DMPA-IM.^14^ More recent online articles and a critical editorial with nine associated online response letters to date in the *British Medical Journal* do all, however, express substantial concerns regarding both the interpretation of the ECHO trial results and the resulting recent changes in the WHO MEC guidelines.^69–71^

Reasons for misreporting are unclear but may be related to some of the statements made in *The Lancet* article, such as the use of the word “safe” in the summary of interpretation of findings.^7^ The statement that “These results support continued and increased access to these three contraceptive methods, as well as expanded contraceptive choices, complemented by high-quality HIV and sexually transmitted infection prevention services”, made under the section “Implications of all the available evidence”,^7^ is also potentially problematic. The authors are, thus, advocating not only continued but also increased access to DMPA-IM, without warning or restriction, which would include for girls and young women, discordant couples, and women in areas with very high HIV incidence and prevalence. This statement appears to have influenced the review group, setting the new WHO guidelines for DMPA-IM and copper-IUD.^6^ The statement also implies that the ECHO trial has disproved the past 30 years worth of accumulated clinical, biological, and *in vitro* evidence on DMPA-IM, and has provided a definitive answer and recommendation for public health policy that we no longer need to have any concerns about DMPA-IM and HIV risk. Clearly, this is not the case.

One potential consequence of misreporting is that there may be little incentive to increase “methods mix” to include more expensive or less widely available methods. Further research on biological effects of MPA or other forms of hormonal contraception could decline due to lack of funding and/or resistance or skeptical queries as to relevance by editors, reviewers, and colleagues. Research to develop new contraceptive methods could suffer, as well as trust in scientists and global health bodies. Most importantly, if there is an increased HIV risk with DMPA-IM relative to no contraception and LNG implant, millions of girls and women may continue to be exposed to more risk than they are aware of, or would choose, and be under a false sense of certainty and safety.

The authors state that: “Indeed, for women desiring effective contraception, the salient question is weighing the relative risks and benefits of different methods, not no method.” This only holds if one assumes that women desire contraception irrespective of HIV risk relative to no contraception or condoms only. One cannot presume that women will still choose to use certain contraceptives if there is an absolute risk that they are not willing to take. They may make other choices. It is surely not the role of scientists to decide on these issues, but rather to present results accurately and objectively so that women, ministries of health, policy makers, and providers can make informed choices. These stakeholders have a right to know the truth. Naturally, all stakeholders desire certainty, but a key question is whether they would rather have false certainty and misinformation than to live with uncertainty. These are important issues that the ECHO results and subsequent press and media releases raise.

In conclusion, although the ECHO study was a well-conducted study with multiple important findings unrelated to HIV acquisition, it did not inform on the absolute HIV risk associated with any one contraceptive method. The meta-analysis of observational data for DMPA-IM relative to no contraception or mainly condom use are currently the best estimate of the HIV risks associated with DMPA-IM and are mostly consistent with a large body of other clinical, animal, and laboratory studies indicating several plausible biological mechanisms whereby DMPA-IM may increase HIV acquisition relative to no contraception.^37–52,72^ The ECHO trial results on relative HIV risks between the three methods are subject to several limitations and uncertainties. They provide evidence that the LNG implant may be associated with lower HIV risk than DMPA-IM and the copper-IUD. The recent decision by the WHO to change the MECs for DMPA-IM and the copper-IUD from category 2 to category 1 based primarily on the ECHO trial results seems ill-considered and unduly hasty. The challenging question remains as to a way forward. It is unlikely that future randomized clinical trials on HIV risk for DMPA-IM will be conducted in high-risk HIV areas, given cost and possibly ethical considerations. The hope is that new and ongoing research and combined evidence from animal, biological, and clinical data accumulated over decades will inform policies that acknowledge uncertainties, likely and theoretical risks and consequences, and stimulate real expansion of contraceptive alternatives^10^ with women's health as the main priority.

## References

[1] United Nations Department of Economic and Social Affairs Population Division: Trends in contraceptive use worldwide 2015 (ST/ESA/SER.A/349). New York: United Nations, 2015

[2] Pfizer: Package leaflet: Information for the user Depo-Provera^®^ 150 mg/ml suspension for injection. United Kingdom: Pfizer Limited, 2016

[3] United Nations, Department of Economic and Social Affairs, Population Division: Estimates and projections of family planning indicators 2019. United Nations, New York, Available at www.un.org/en/development/desa/population/theme/family-planning/cp_model.asp (2019), accessed 91, 2019

[4] Polis CB, Curtis KM, Hannaford PC, *et al.*: An updated systematic review of epidemiological evidence on hormonal contraceptive methods and HIV acquisition in women. AIDS 2016;30:2665–2683 27500670 10.1097/QAD.0000000000001228 PMC5106090

[5] Morrison CS, Chen PL, Kwok C, *et al.*: Hormonal contraception and the risk of HIV acquisition: An individual participant data meta-analysis. PLoS Med 2015;12:e1001778 25612136 10.1371/journal.pmed.1001778 PMC4303292

[6] World Health Organization: Contraceptive eligibility for women at high risk of HIV. Guidance statement: Recommendations on contraceptive methods used by women at high risk of HIV. Available at https://apps.who.int/iris/bitstream/handle/10665/326653/9789241550574-eng.pdf;jsessionid=42DF3CFFEC6DF720ABBDFA3A74FA2573?ua=1 (2019), accessed 830, 2019 31503432

[7] Evidence for Contraceptive Options HIV Outcomes (ECHO) Trial Consortium: HIV incidence among women using intramuscular depot medroxyprogesterone acetate, a copper intrauterine device, or a levonorgestrel implant for contraception: A randomised, multicentre, open-label trial. Lancet 2019;394:303–313 31204114 10.1016/S0140-6736(19)31288-7 PMC6675739

[8] Jones HE: Time to focus on improving the contraceptive method mix in high HIV prevalence settings and let go of unanswerable questions. Contraception 2014;90:357–359 24993486 10.1016/j.contraception.2014.05.014

[9] Rees H; ECHO Consortium: DMPA and HIV: Why we need a trial. Contraception 2014;90:354–356 25183263 10.1016/j.contraception.2014.08.007

[10] Gollub EL, Jones HE, Ralph LJ, van de Wijgert JHHM, Padian N, Stein Z: The need for policy change regarding progestin-only injectable contraceptives. J Womens Health (Larchmt) 2019;28:1180–1184 30576259 10.1089/jwh.2018.7284 PMC6909683

[11] Sathyamala C: In the name of science: Ethical violations in the ECHO randomised trial. Glob Public Health 2019;1–16. 10.1080/17441692.2019.1634118 31234717

[12] TimesLIVE: SA's most popular contraceptive injection does not increase HIV risk. Available at www.timeslive.co.za/news/sci-tech/2019-06-13-sas-most-popular-contraceptive-injection-does-not-increase-hiv-risk (2019), accessed 614, 2019

[13] Bhekisisa Centre for Health Journalism: The ECHO trial is out: Depo Provera doesn't lead to HIV. Available at https://bhekisisa.org/multimedia/2019-06-13-echo-trial-results-contraception-lancet-depo-provera-dmpa-family-planning (June 13, 2019) accessed 614, 2019

[14] The New York Times: Depo-Provera, an injectable contraceptive, does not raise H.I.V. risk. Available at www.nytimes.com/2019/06/13/health/depo-provera-hiv-africa.html (June 13, 2019), accessed 614, 2019

[15] Daily Maverick Health-e News: ECHO trial results: Now we know, but this is not a victory. Available at www.dailymaverick.co.za/article/2019-06-14-echo-trial-results-now-we-know-but-this-is-not-a-victory (June 14, 2019), accessed 87, 2019

[16] Daily Maverick Health-e News: Inside the ECHO chamber: Government must prioritise reproductive justice. Available at www.dailymaverick.co.za/article/2019-06-30-inside-the-echo-chamber-government-must-prioritise-reproductive-justice (June 30, 2019) accessed 87, 2019

[17] WHO Sexual and Reproductive Health: New study finds no link between HIV infection and contraceptive methods. Available at www.who.int/reproductivehealth/hc-hiv/en (June 13, 2019), accessed 614, 2019

[18] ECHO: ECHO finds no substantial difference in HIV risk among DMPA-IM, copper IUD, and LNG implant users. Available at http://echo-consortium.com/wp-content/uploads/2019/06/CONFIDENTIAL_ECHO_release_13June2019_FINAL-2.pdf (June 13, 2019), accessed 614, 2019

[19] Healio Infectious Disease News: ECHO trial: HIV risk does not differ by contraceptive method. Available at: www.healio.com/infectious-disease/hiv-aids/news/online/%7B295a17f0-8869-4668-9383-14507320006a%7D/echo-trial-hiv-risk-does-not-differ-by-contraceptive-method (June 13, 2019), accessed 87, 2019

[20] Altman DG, Bland JM: Absence of evidence is not evidence of absence. BMJ 1995;311:485 7647644 10.1136/bmj.311.7003.485 PMC2550545

[21] Wasserstein RL, Schirm AL, Lazar NA: Moving to a world beyond “p < 0.05.” Am Stat 2019;73:1–19

[22] Amrhein V, Greenland S, McShane B: Scientists rise up against statistical significance. Nature 2019;567:305–307 30894741 10.1038/d41586-019-00857-9

[23] Goodman SN: How sure are you of your result? Put a number on it. Nature 2018;564:7 30514950 10.1038/d41586-018-07589-2

[24] Editorial: It's time to talk about ditching statistical significance. Nature 2019;567:283 10.1038/d41586-019-00874-8 30894740

[25] Hurlbert SH, Levine RA, Utts J: Coup de Grâce for a tough old bull: “Statistically significant” expires. Am Stat 2019;73:352–357

[26] Altman N, Krzywinski M: Interpreting P values. Nat Methods 2017;14:213

[27] Greenland S: Nonsignificance plus high power does not imply support for the null over the alternative. Ann Epidemiol 2012;22:364–368 22391267 10.1016/j.annepidem.2012.02.007

[28] Hoenig JM, Heisey DM: The abuse of power. Am Stat 2001;55:19–24

[29] Hofmeyr GJ, Morrison CS, Baeten JM, *et al.*: Rationale and design of a multi-center, open-label, randomised clinical trial comparing HIV incidence and contraceptive benefits in women using three commonly-used contraceptive methods (the ECHO study). Gates Open Res 2017;1:17 29355224 10.12688/gatesopenres.12775.1 PMC5771152

[30] UNAIDS: UNAIDS data 2018. Available at: www.unaids.org/en/resources/documents/2018/unaids-data-2018 (2018), accessed 66, 2019

[31] Butler AR, Smith JA, Polis CB, Gregson S, Stanton D, Hallett TB: Modelling the global competing risks of a potential interaction between injectable hormonal contraception and HIV risk. AIDS 2013;27:105–113 23014519 10.1097/QAD.0b013e32835a5a52 PMC4862571

[32] National Department of Health (NDoH), Statistics South Africa (Stats SA), South African Medical Research Council (SAMRC), ICF: South Africa demographic and health survey 2016. NDoH, Stats SA, SAMRC, and ICF, Pretoria, South Africa, Rockville, MD. Available at http://dhsprogram.com/pubs/pdf/FR337/FR337.pdf (2019), accessed 614, 2019

[33] Beacroft L, Smith JA, Hallett TB: What impact could DMPA use have had in South Africa and how might its continued use affect the future of the HIV epidemic? J Int AIDS Soc 2019;22:e25414 31729195 10.1002/jia2.25414 PMC6856612

[34] Hernan MA, Hernandez-Diaz S, Robins JM: Randomized trials analyzed as observational studies. Ann Intern Med 2013;159:560–562 24018844 10.7326/0003-4819-159-8-201310150-00709 PMC3860874

[35] Heffron R, Parikh UM, Penrose KJ, *et al.*: Objective measurement of inaccurate condom use reporting among women using depot medroxyprogesterone acetate for contraception. AIDS Behav 2017;21:2173–2179 27699594 10.1007/s10461-016-1563-y PMC5378697

[36] Achilles SL, Mhlanga FG, Musara P, Poloyac SM, Chirenje ZM, Hillier SL: Misreporting of contraceptive hormone use in clinical research participants. Contraception 2018;97:346–353 28966052 10.1016/j.contraception.2017.09.013 PMC5858917

[37] Thurman A, Chandra N, Schwartz JL, *et al.*: The effect of hormonal contraception on cervicovaginal mucosal end points associated with HIV acquisition. AIDS Res Hum Retroviruses 2019;35:853–864 30997816 10.1089/AID.2018.0298

[38] Maritz MF, Ray RM, Bick AJ, *et al.*: Medroxyprogesterone acetate, unlike norethisterone, increases HIV-1 replication in human peripheral blood mononuclear cells and an indicator cell line, via mechanisms involving the glucocorticoid receptor, increased CD4/CD8 ratios and CCR5 levels. PLoS One 2018;13:e0196043 29698514 10.1371/journal.pone.0196043 PMC5919616

[39] Ray RM, Maritz MF, Avenant C, *et al.*: The contraceptive medroxyprogesterone acetate, unlike norethisterone, directly increases R5 HIV-1 infection in human cervical explant tissue at physiologically relevant concentrations. Sci Rep 2019;9:4334 30867477 10.1038/s41598-019-40756-7 PMC6416361

[40] Zalenskaya IA, Chandra N, Yousefieh N, *et al.*: Use of contraceptive depot medroxyprogesterone acetate is associated with impaired cervicovaginal mucosal integrity. J Clin Invest 2018;128:4622–4638 30222141 10.1172/JCI120583 PMC6159996

[41] Hapgood JP, Kaushic C, Hel Z: Hormonal contraception and HIV-1 acquisition: Biological mechanisms. Endocr Rev 2018;39:36–78 29309550 10.1210/er.2017-00103 PMC5807094

[42] Quispe Calla NE, Vicetti Miguel RD, Glick ME, Kwiek JJ, Gabriel JM, Cherpes TL: Exogenous oestrogen inhibits genital transmission of cell-associated HIV-1 in DMPA-treated humanized mice. J Int AIDS Soc 2018;21:e25063 10.1002/jia2.25063 PMC5810324 29334191

[43] Smith-McCune KK, Hilton JF, Shanmugasundaram U, *et al.*: Effects of depot-medroxyprogesterone acetate on the immune microenvironment of the human cervix and endometrium: Implications for HIV susceptibility. Mucosal Immunol 2017;10:1270–1278 28051087 10.1038/mi.2016.121 PMC5496803

[44] Dabee S, Barnabas SL, Lennard KS, *et al.*: Defining characteristics of genital health in South African adolescent girls and young women at high risk for HIV infection. PLoS One 2019;14:e0213975 30947260 10.1371/journal.pone.0213975 PMC6448899

[45] Morrison CS, Fichorova R, Chen PL, *et al.*: A longitudinal assessment of cervical inflammation and immunity associated with HIV-1 infection, hormonal contraception, and pregnancy. AIDS Res Hum Retroviruses 2018;34:889–899 30047279 10.1089/aid.2018.0022 PMC6204564

[46] Li L, Zhou J, Wang W, *et al.*: Effects of three long-acting reversible contraceptive methods on HIV target cells in the human uterine cervix and peripheral blood. Reprod Biol Endocrinol 2019;17:26 30795774 10.1186/s12958-019-0469-8 PMC6387540

[47] Lajoie J, Tjernlund A, Omollo K, *et al.*: Increased cervical CD4(+)CCR5(+) T cells among Kenyan sex working women using depot medroxyprogesterone acetate. AIDS Res Hum Retroviruses 2019;35:236–246 30585733 10.1089/aid.2018.0188 PMC6434599

[48] Piccinni MP, Lombardelli L, Logiodice F, Kullolli O, Maggi E, Barkley MS: Medroxyprogesterone acetate decreases Th1, Th17, and increases Th22 responses via AHR signaling which could affect susceptibility to infections and inflammatory disease. Front Immunol 2019;10:642 31001262 10.3389/fimmu.2019.00642 PMC6456711

[49] Woods MW, Zahoor MA, Dizzell S, Verschoor CP, Kaushic C Medroxyprogesterone acetate-treated human, primary endometrial epithelial cells reveal unique gene expression ignature linked to innate immunity and HIV-1 susceptibility. Am J Reprod Immunol Jan 2018;79 DOI: 10.1111/aji.12781 29105931

[50] Tasker C, Davidow A, Roche NE, Chang TL: Depot medroxyprogesterone acetate administration alters immune markers for HIV preference and increases susceptibility of peripheral CD4(+) T cells to HIV infection. Immunohorizons 2017;1:223–235 29188238 10.4049/immunohorizons.1700047 PMC5703073

[51] Polis CB, Achilles SL, Hel Z, Hapgood JP: Is a lower-dose, subcutaneous contraceptive injectable containing depot medroxyprogesterone acetate likely to impact women's risk of HIV? Contraception 2018;97:191–197 29242082 10.1016/j.contraception.2017.12.003 PMC5828886

[52] Heffron R, Achilles SL, Dorflinger LJ, *et al.*: Pharmacokinetic, biologic and epidemiologic differences in MPA- and NET-based progestin-only injectable contraceptives relative to the potential impact on HIV acquisition in women. Contraception 2019;99:199–204 30576636 10.1016/j.contraception.2018.12.001 PMC6467541

[53] Abaasa A, Asiki G, Price MA, *et al.*: Comparison of HIV incidence estimated in clinical trial and observational cohort settings in a high risk fishing population in Uganda: Implications for sample size estimates. Vaccine 2016;34:1778–1785 26923456 10.1016/j.vaccine.2016.02.048 PMC4994889

[54] World Health Organization: Medical eligibility criteria for contraceptive use, 5th edition. Available at www.who.int/reproductivehealth/publications/family_planning/MEC-5/en (2015), accessed 614, 2019 26447268

[55] Quispe Calla NE, Vicetti Miguel RD, Boyaka PN, *et al.*: Medroxyprogesterone acetate and levonorgestrel increase genital mucosal permeability and enhance susceptibility to genital herpes simplex virus type 2 infection. Mucosal Immunol 2016;9:1571–1583 27007679 10.1038/mi.2016.22 PMC5035233

[56] Hickey M, Marino JL, Tachedjian G: Critical review: Mechanisms of HIV transmission in Depo-Provera users: The likely role of hypoestrogenism. J Acquir Immune Defic Syndr 2016;71:1–7 26761267 10.1097/QAI.0000000000000805

[57] Pereda MD, Farina SB, Fernandez Lorenzo M: Is the early fragmentation of intrauterine devices caused by stress corrosion cracking? Acta Biomater 2009;5:3240–3246 19447217 10.1016/j.actbio.2009.04.033

[58] Mastro MA, Hardy AW, Boasso A, Shearer GM, Eddy CR, Kub FJ: Non-toxic inhibition of HIV-1 replication with silver–copper nanoparticles. Med Chem Res 2010;19:1074–1081

[59] Ammala M, Nyman T, Strengell L, Rutanen EM: Effect of intrauterine contraceptive devices on cytokine messenger ribonucleic acid expression in the human endometrium. Fertil Steril 1995;63:773–778 7890061 10.1016/s0015-0282(16)57480-9

[60] Sheppard BL: Endometrial morphological changes in IUD users: A review. Contraception 1987;36:1–10 10.1016/0010-7824(87)90057-6 3117492

[61] Shobokshi A, Shaarawy M: Cervical mucus granulocyte macrophage colony stimulating factor and interleukin-2 soluble receptor in women using copper intrauterine contraceptive devices. Contraception 2002;66:129–132 12204787 10.1016/s0010-7824(02)00331-1

[62] Woolley JA, Seleem S, Hills FA, Salem H, el-Nashar E, Chard T: Raised circulating levels of interleukin-6 in women with an intrauterine contraceptive device. Gynecol Obstet Invest 1996;42:241–243 8979095 10.1159/000291972

[63] Heffron R, Donnell D, Rees H, *et al.*: Use of hormonal contraceptives and risk of HIV-1 transmission: A prospective cohort study. Lancet Infect Dis 2012;12:19–26 21975269 10.1016/S1473-3099(11)70247-X PMC3266951

[64] Baeten JM, Benki S, Chohan V, *et al.*: Hormonal contraceptive use, herpes simplex virus infection, and risk of HIV-1 acquisition among Kenyan women. AIDS 2007;21:1771–1777 17690576 10.1097/QAD.0b013e328270388a

[65] Crook AM, Ford D, Gafos M, *et al.*: Injectable and oral contraceptives and risk of HIV acquisition in women: An analysis of data from the MDP301 trial. Hum Reprod 2014;29:1810–1817 24838704 10.1093/humrep/deu113 PMC4093991

[66] Koubovec D, Ronacher K, Stubsrud E, Louw A, Hapgood JP: Synthetic progestins used in HRT have different glucocorticoid agonist properties. Mol Cell Endocrinol 2005;242:23–32 16125839 10.1016/j.mce.2005.07.001

[67] Huijbregts RP, Michel KG, Hel Z: Effect of progestins on immunity: Medroxyprogesterone but not norethisterone or levonorgestrel suppresses the function of T cells and pDCs. Contraception 2014;90:123–129 24674041 10.1016/j.contraception.2014.02.006 PMC4874781

[68] Govender Y, Avenant C, Verhoog NJ, *et al.*: The injectable-only contraceptive medroxyprogesterone acetate, unlike norethisterone acetate and progesterone, regulates inflammatory genes in endocervical cells via the glucocorticoid receptor. PLoS One 2014;9:e96497 24840644 10.1371/journal.pone.0096497 PMC4026143

[69] AVAC: Global Advocacy for HIV prevention. AVAC's Take on Updated WHO Guidance on Hormonal Contraception and HIV Risk. Available at www.avac.org/blog/updated-who-guidance-hc-hiv-risk (August 29 2019), accessed 830, 2019

[70] Green A, Pilane P: New WHO contraceptive guidelines “disturbing.” Health-e News. Available at https://health-e.org.za/2019/08/29/world-health-organization-guidelines-contraceptives-depo-provera-echo-trial (August 29, 2019), accessed 830, 2019

[71] Sathyamala C: Depot contraception and HIV: An exercise in obfuscation. BMJ 2019;367:l5768 31591088 10.1136/bmj.l5768

[72] Wessels JM, Lajoie J, Cooper M, *et al.*: Medroxyprogesterone acetate alters the vaginal microbiota and microenvironment in women and increases susceptibility to HIV-1 in humanized mice. Dis Model Mech Oct 23 2019;12 DOI: 10.1242/dmm.039669 PMC6826019 31537512

